# What next for gain-of-function research in Europe?

**DOI:** 10.7554/eLife.13035

**Published:** 2015-12-30

**Authors:** Robin Fears, Volker ter Meulen

**Affiliations:** 1 European Academies Science Advisory Council, Halle, Germany; 2 German National Academy of Sciences Leopoldina, Halle, Germany

**Keywords:** science policy, risk-benefit analysis, biosafety and biosecurity, bioethics, gain-of-function research, influenza

## Abstract

A working group on gain-of-function research set up by the European Academies Science Advisory Council (EASAC) has emphasised the importance of ensuring that the necessary safeguards and policies are in place

Infectious diseases continue to be responsible for a substantial proportion of deaths worldwide and there is evidence for a significant increase in the number of outbreaks over the past 30 years. Although some of this increase may be due to improved detection and reporting, animal diseases that can be transmitted to humans are among the major causes of human infection (Smith et al., 2014).

Influenza outbreaks are a particular concern: in the UK, for example, the potential impact of an influenza pandemic has been identified as the highest priority in the UK Government Risk Register for 2015. Our current inability to predict which specific virus subtypes will trigger the next influenza pandemic highlights the vital importance of addressing gaps in the knowledge base. Research on a wide range of topics—including the study of virus transmission, host range, drug resistance, infectivity, immunity and virulence—is urgently needed to fill these gaps. In this article we focus on the issues raised by proposals to use ‘gain-of-function’ experiments to fill some of these gaps. Such experiments have a long history of providing useful information in virology.

Gain-of-function experiments involve modifying a virus and analysing the link between modified genotype and phenotype. However, proposals to modify the H5N1 variant of the influenza virus in order to affect its transmission potential, and thereby understand the factors that determine the ability of an animal virus to spread to humans (and between humans) by the aerosol route, were controversial (Russell et al., 2014; Schultz-Cherry et al., 2014; Palu, 2014; Wain-Hobson, 2014), and there is still a de facto moratorium on such research in the US.

Self-regulation is a necessary first step for gain-of-function research, but this does not mean that each researcher is free to decide for themselves what procedures to follow.

Experiments are of concern if they have the potential to cause serious disease, and these concerns encompass biosafety (relating to the accidental release of a pathogen from containment), biosecurity (relating to intentional misuse; see NRC, 2004), and bioethics (the principles and their place in research review procedures). Although potentially dangerous research studies are already subject to stringent regulations in many countries, recent controversies prompted the European Academies Science Advisory Council (EASAC) to set up a working group to examine the issues raised by gain-of-function research and to make recommendations for the management of such research and its outputs in the EU. This working group contained the present authors and other scientists with a wide range of relevant experience. Here we discuss some of the recommendations made by the working group (see EASAC, 2015 for the full report).

## Scientific responsibility and benefit-risk assessment

Self-regulation is a necessary first step for gain-of-function research, but this does not mean that each researcher is free to decide for themselves what procedures to follow. Everyone involved must conform to existing regulations, codes of conduct and established methods for biorisk management (see EASAC, 2015 for a list of existing regulations in Europe, and details of work by the World Health Organisation and the OECD in this area).

Moreover, proposals for gain-of-function studies—particularly those that intend to alter virus transmission, host range, drug resistance, infectivity, immunity and virulence—have to be justified by their authors to a wide range of bodies (the institutions where the work will be carried out, the agencies that will fund the work, the relevant ethics committees, and the relevant national authorities), as well as their peers, on a case-by-case basis. This process must involve a thorough analysis of the risks and benefits of the research being proposed. Researchers also need to demonstrate that the information they need can only be obtained from gain-of-function experiments.

A clear theme in both Europe and the US is the need for the scientific community to engage more broadly with the public, explaining the reasons for doing gain-of-function research.

There are many uncertainties in the data used to evaluate benefit and risk, and this can lead to controversy. It should also be acknowledged that potential benefits of research are sometimes overstated by scientists: however, it is also true that the benefits of research might only become clear much later. Views vary on whether benefit should be quantified in terms of future public health impact or described in terms of the generation of knowledge, and on the extent to which benefits may be lost if research is not allowed. There have been concerns that research that could help to streamline the production of vaccines might suffer if gain-of-function experiments are banned (Cohen, 2015; Ping et al., 2015). Because of the multiple challenges involved in assessing risk and benefit, the EASAC report concludes that any benefit-risk analysis cannot be regarded as a ‘once and for all’ calculation, and that all the relevant parties need to understand and communicate the issues on an on-going basis.

The EASAC report also recommends that there is no need for a new advisory body at the EU level. Rather, all EU Member States should have a clear national advisory mechanism on procedures for assessing and managing biosafety and biosecurity risks. In the UK, for example, the Health and Safety Executive has statutory powers, but other EU Member States have different mechanisms and not all have statutory powers. There is need to harmonise the implementation of good practice in these respects. All countries should also adopt a ‘layered’ approach with researchers, research institutions, research funders and national authorities all being responsible for the regulation of gain-of-function research. Such a layered approach at the country level will spread good practice, increase accountability, and help to improve public trust in research.

Researchers and institutions also have to recognise their responsibility to make decisions about publishing sensitive information and, together with funders, ethics committees and others, need to consider these issues throughout the research process, starting when the research is first proposed, rather than delaying the decision until a manuscript is ready for submission to a journal. The oversight within the scientific community must involve journals and professional societies, as well as researchers, their funders and institutions (and national advisory bodies in complex cases). Journal editors in the US recently came to a similar conclusion (Casadevall et al., 2015). Regarding the question of what to do when European researchers submit a manuscript to a journal that is not based in Europe, EASAC advises that the use of the EU’s export control regime is an inappropriate and ineffective vehicle to block such submissions.

Many of EASAC’s recommendations have been welcomed by the European Commission, who will now consider how to incorporate EASAC advice into guidance for Horizon 2020 research grant applicants and evaluators.

The EASAC report resonates with the themes that are emerging from discussions in the US involving the National Academies, the National Science Advisory Board for Biosecurity (NSABB) and the National Institutes of Health. A National Academies workshop in December 2014 observed that the challenges involved in regulating gain-of-function research were international, that attention should be focussed on those experiments of greatest concern, that researchers and their institutions must accept responsibility, and that analysing the risks and benefits is not straightforward. Since then, a quantitative risk-benefit analysis has been initiated (Casagrande et al., 2015), and the NSABB has set up a working group to look into the issues surrounding gain-of-function research (Kanabrocki, 2015). In its interim report the NASBB working group noted that the US already has a robust policy framework for the regulation of research, and any future policies should build on this framework.

## Challenges for wider engagement

A clear theme in both Europe and the US is the need for the scientific community to engage more broadly with the public, explaining the reasons for doing gain-of-function research, discussing the potential risks and benefits of this research, and explaining the biorisk management practices adopted. Engagement with public interests can be considered at three levels. First, on the global scale, the scientific community has to ensure that the public health benefits that arise from innovation based on gain-of-function research are made available to everyone. Second, on the national level, scientists need to convince the public that taxpayer funds are being spent wisely. Third, on a local level, scientists must engage with those who live near research facilities to reassure them about safety.

Another clear theme is the need for the regulatory authorities, funding bodies and professional societies in different countries to work together to share expertise in the regulation of gain-of-function research and in risk-benefit analysis. Academies of science have a clear role to play at the national level, and international networks of academies can bring scientists from different countries together to study and make recommendations on specific issues: the working group on biosecurity set up by the InterAcademy Partnership is an example of this.

Influenza pandemics are currently unpredictable. However, we still need to be ready for the next pandemic, and that includes being in a position to perform gain-of-function research if that is the only way to obtain the information needed to deal with the pandemic. And, as should be clear from above, this means that many different stakeholders—scientists, institutions, funding agencies, ethics committees, national regulatory authorities, scientific societies and journals—need to work together to ensure that we are prepared.
